# Analysis of fine particulates from fuel burning in a reconstructed building at Çatalhöyük World Heritage Site, Turkey: assessing air pollution in prehistoric settled communities

**DOI:** 10.1007/s10653-021-01000-2

**Published:** 2021-06-21

**Authors:** Lisa-Marie Shillito, Anil Namdeo, Aishwarya Vikram Bapat, Helen Mackay, Scott D. Haddow

**Affiliations:** 1 School of History, Classics and Archaeology, Newcastle University, Newcastle upon Tyne, UK; 2 Department of Geography and Environmental Sciences, Northumbria University, Newcastle upon Tyne, UK; 3 School of Engineering, Newcastle University, Newcastle upon Tyne, UK; 4 Department of Geography, Durham University, Durham, UK; 5 Department of Cross-Cultural and Regional Studies, Copenhagen University, Copenhagen, Denmark

**Keywords:** Çatalhöyük, Neolithic, Air quality monitoring, Biofuel, PM_2.5_ pollution

## Abstract

The use of wood, dung and other biomass fuels can be traced back to early prehistory. While the study of prehistoric fuel use and its environmental impacts is well established, there has been little investigation of the health impacts this would have had, particularly in the Neolithic period, when people went from living in relatively small groups, to living in dense settlements. The UNESCO World Heritage Site of Çatalhöyük, Turkey, is one of the earliest large ‘pre-urban’ settlements in the world. In 2017, a series of experiments were conducted to measure fine particulate (PM_2.5_) concentrations during typical fuel burning activities, using wood and dung fuel. The results indicate that emissions from both fuels surpassed the WHO and EU standard limits for indoor air quality, with dung fuel being the highest contributor for PM_2.5_ pollution inside the house, producing maximum values > 150,000 µg m^−3^. Maximum levels from wood burning were 36,000 µg m^−3^. Average values over a 2–3 h period were 13–60,000 µg m^−3^ for dung and 10–45,000 µg m^−3^ for wood. The structure of the house, lack of ventilation and design of the oven and hearth influenced the air quality inside the house. These observations have implications for understanding the relationship between health and the built environment in the past.

**Supplementary Information:**

The online version contains supplementary material available at 10.1007/s10653-021-01000-2.

## Introduction

Air pollution is often associated with industrialisation in the nineteenth century, but the origins of anthropogenic air pollution can be seen much earlier. The earliest evidence for exposure to pollutants is seen in Neanderthals at the Spanish cave site of El Sidron where chemical signals in teeth show smoke inhalation from campfires around 49,000 years ago (Hardy et al., 2012). Early hominid populations were the first to manipulate fire for warmth and cooking (Berna et al., 2012), an ability that accelerated with permanent settlement and the development of pyrotechnologies. The development of pottery production, followed by early metal working and eventually for more sophisticated processes such as glass making, all involved the use of fuels, increasing people’s exposure to by-products of smoke and other pollutants (Makra & Brimblecombe, 2004).

**Fig. 1:**
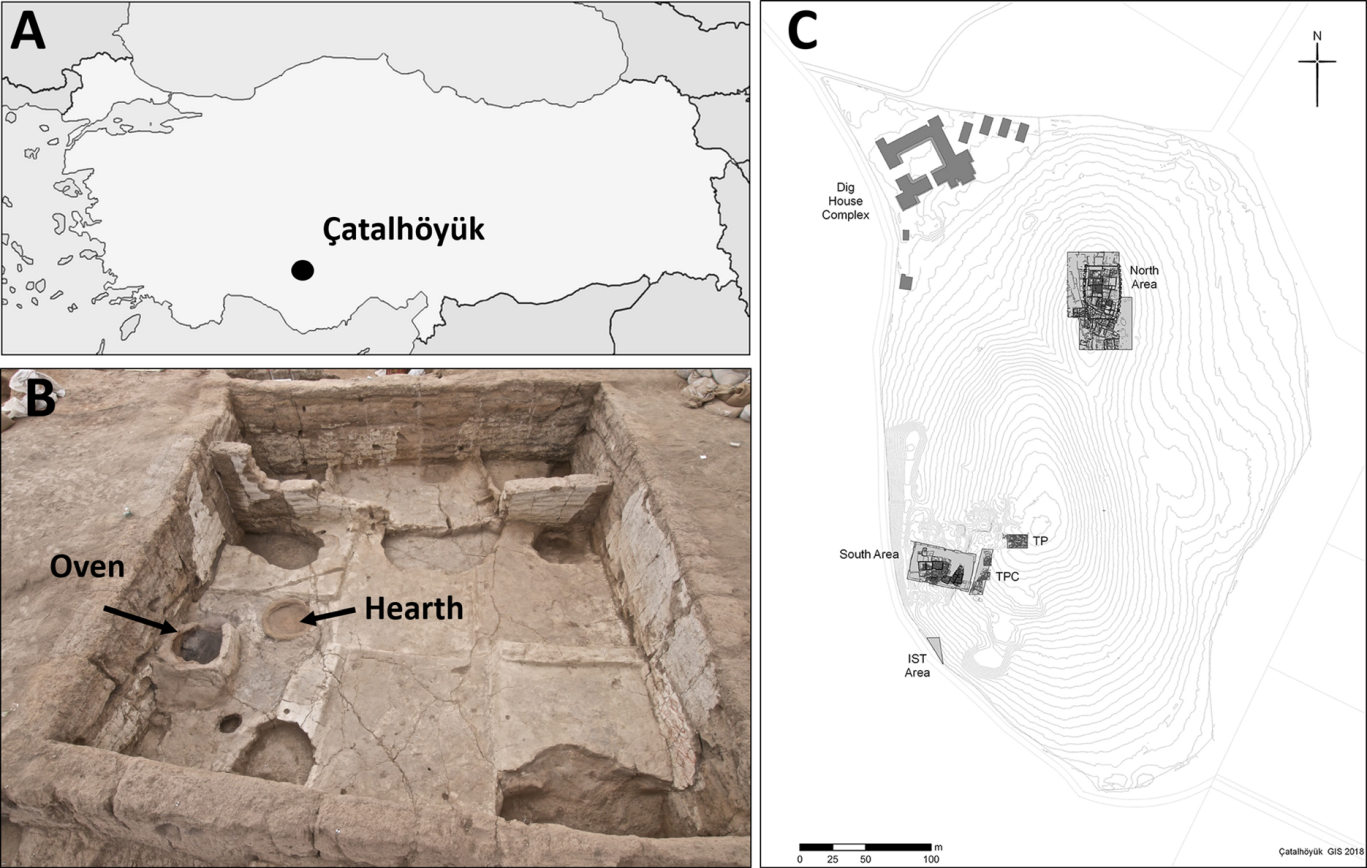
**A** Map showing the location of Çatalhöyük in Turkey. Outline map from Wikimedia commons. **B** Building 119 in North Area, excavated 2014, showing a typical example of an oven and hearth. Photo courtesy of Çatalhöyük project CC BY-NC-SA 2.0. **C** Plan of the site showing excavation areas. Plan by Camilla Mazucatto

Çatalhöyük is a UNESCO World Heritage Site, and is an ideal case study for investigating relationships between health and the built environment in prehistory. The site has > 1000 years of continuous occupation from the pre-pottery Neolithic to Chalcolithic period 7100–5700 BC (Bayliss et al., 2015). At its peak, over 8000 people lived there. Each level of occupation at Çatalhöyük contains multiple mudbrick buildings ranging in size from 15 to 25 m^2^, clustered into ‘neighbourhoods’. Large open areas between neighbourhood clusters contain substantial build-up of midden deposits and ‘bonfire’ deposits (Shillito, 2011). A typical building has a central room with an oven and hearth, and slightly elevated platforms (Fig. 1). The interior of these rooms, including the platforms, is coated with white plaster, and microscopic observations have shown layer of soot and frequent re-plastering (Matthews, 2005a, 2005b). The buildings are estimated to have been in use for around 70 years before being dismantled and rebuilt in the same location. These processes of rebuilding occur multiple times over the site’s occupation (Cessford, 2005; Düring, 2005). The Çatalhöyük settlement changes from a dense agglomeration in its earliest phase, where individual buildings are constructed wall to wall with no gaps or streets between them, to a more open nucleated settlement towards the end of the occupation.

A wide range of materials have been used as fuels in prehistory including wood, animal dung, reeds, peat/turf and agricultural waste products (Braadbaart et al., 2017). While wood is often used, in some cases, non-wood fuels were preferentially selected for particular activities, e.g. dung for pottery production, as it burns more evenly and slowly than wood (Sillar, 2000). Research at Çatalhöyük and other sites in Anatolia suggests that non-wood fuels, particularly *Phragmites* reeds and animal dung, became more important over time (Portillo et al., 2020; Shillito et al., 2011). There is also evidence that certain fuel burning activities were located outdoors (Bogaard et al., 2013; Shillito & Ryan, 2013), and that dung is associated more with outdoor burning activities (Portillo et al., 2020). Given the large volume of fuel by-product deposits and the ubiquity of fuel burning activities, it is highly likely that the inhabitants were exposed to air pollutants from burning activities. High concentrations of silica particles in the form of phytoliths from grasses and reeds have also been identified in fuel deposits (Portillo et al., 2020; Shillito et al., 2011), which are known in historic and modern contexts to cause silicosis, a lung disease caused by inhalation of silica dust (Köksal & Kahraman., 2011; Akgün et al., 2015).

We know from modern studies that burning ‘biofuels’ has significant negative consequences on health, especially in enclosed spaces with poor ventilation, but the relationship between fuel use and health in prehistory has never been explored. Studies of health in the archaeological record are typically focussed on the analysis of human skeletal remains. There are several indicators of respiratory disease that can be detected in the osteoarchaeological record, for example inflammation of the maxillary sinuses, and lesions on the visceral surfaces of ribs, which can be linked to diseases such as tuberculosis, chronic bronchitis, pneumonia and cancer (Davies-Barrett et al., 2019; Roberts, 2007, 2015). While there are some examples of Neolithic populations with skeletal indicators of respiratory disease (Sparacello et al., 2016, 2017), respiratory diseases do not always leave such traces on bone. Complicating our interpretation further is the so-called “osteological paradox” (Wood et al., 1992) in which a skeleton with a “healthy” appearance (i.e. free of bony lesions) may indicate an individual who succumbed very quickly to disease before their bones could be affected. Alternatively, individuals with significant skeletal indicators of disease are likely to have lived with their illnesses for an extended period before succumbing.

Recent advances in emission and dispersion modelling in urban environment, coupled with the availability of details time activity patterns and pervasive monitoring of air pollution in our cities can enhance our understanding of individual and community level exposures and disease burdens (Namdeo et al., 2011, 2015). The exceptional preservation of buildings at Çatalhöyük means that there is much potential for applying these modern methodologies to this prehistoric settlement, and to provide estimations of exposure levels that could help better understand the links between the built environment and health in the past. This may provide an alternate line of evidence that can be assessed alongside osteoarchaeological data.

Between 1997–1999 a replica of a Neolithic mudbrick building at Çatalhöyük was constructed to the south of the site’s visitor centre, to provide a visual representation of what one of the houses may have looked like during the time of occupation (Gargett, 2017). A team of archaeologists and local builders from the local village of Küçükköy and Çumra made mudbricks using traditional materials and used these to replicate a ‘typical’ building, as seen in the archaeological record (Stevanović, 1999). Hay was used for a thatched roof and the inside was plastered. The entrance of the house was typically from the roof of the structure, with no windows or doorways (the modern replica has a doorway for practical purposes). Experimental archaeology and replicas of archaeological buildings have been used at several sites as an educational tool for visitors, and these reconstructions are also increasingly used for research purposes (e.g. Rhyl-Svensen et al., 2010; Banerjea et al., 2015). The aim of this research was to measure pollution levels from episodes of fuel use to test how living in these buildings may have impacted the exposure of the inhabitants to fine particulate matter and thus their respiratory health. The focus of the study is PM_2.5_, fine particulate matter with a diameter of 2.5 microns or less. These particles are the most serious health risk in air pollution, as they are small enough to travel deep into the lungs, where they become embedded (Feng et al., 2016).

## Methodology

### Experimental set-up

**Fig. 2:**
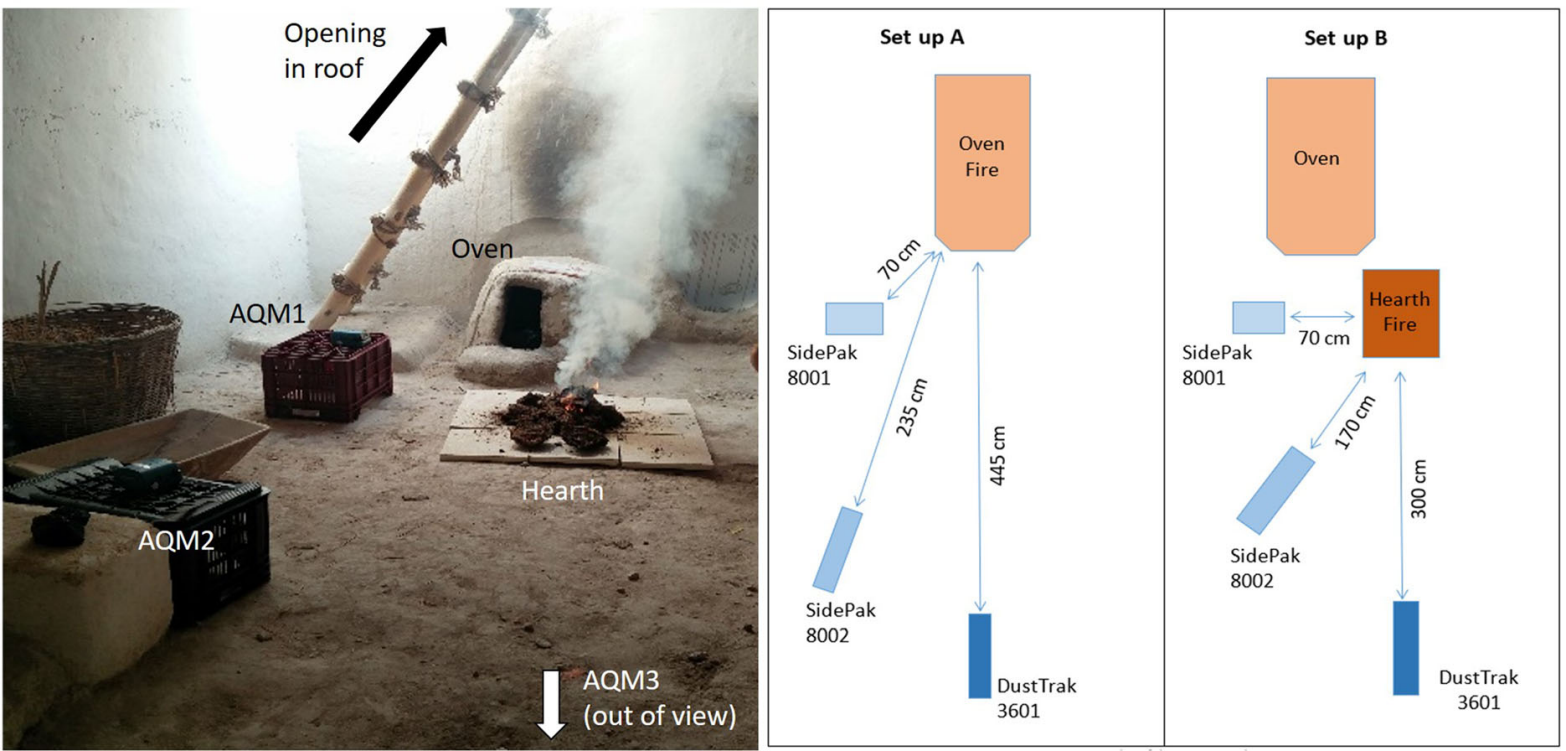
Photograph showing an experiment in progress, and plan of the set-up A (fuel in oven) and set-up B (fuel on hearth area)

The experiments were conducted over three days, 11th, 12th and 13th of July 2017. During this period, the regional temperature averaged 24 °C with no precipitation and calm winds (GMAO 2015). Five tests were conducted to measure the PM_2.5_ concentrations under two different set-ups (Table 1, Fig. 2). Test 1 was set up to assess background dust levels. Set-up ‘A’ (tests, 3, 4 and 5) was designed to measure the PM_2.5_ levels released from the oven, while set-up ‘B’ (test 2) was designed to measure the PM_2.5_ levels due to burning of fuel in the hearth.

**Table 1:** Details of the tests conducted

Test	Set up	Duration (mins)	Fuels used	Fuel location	Purpose
1	–	15	None	–	Background dust measurements
2	B	240	Dung	Hearth	Dung fuel test
3	A	156	Wood	Oven	Wood fuel test
4	A	280	Wood and dung	Oven	Mixed fuel test
5	A	628	Wood and twigs	Oven	Wood fuel test—extended duration

The oven was enclosed from three sides with an opening at the front to load fuel, and another smaller opening at the top to release the smoke produced. The hearth area to the front of the oven was completely open on all sides. Two types of biofuel were selected, wood and dung. The fire was built with the help of local residents from Küçükköy, who supplied the dung and wood. The grass-fed cow dung used in the tests was dried prior to the investigation.

### Fine particulate measurements and data analysis

Three air quality monitoring (AQM) stations were used for the measurement the air pollution inside the house, one TSI SidePak AM510, one TSI SidePak AM520 and one TSI DustTrak 2601 monitor, all three classified as personal aerosol monitors (Table 2). These were set up to monitor PM_2.5_ concentrations and calibrated to zero before the experiments. We used three AQMs to make sure we recorded measurements from different areas around the oven/hearth in the building, both directly in front of the oven/hearth and to each side. The positions of the AQMs from the oven/hearth were chosen as the likely location that people living in the building would be located during ‘typical’ activity in the house.

**Table 2:** Air quality monitoring (AQM) stations used in this study

Air quality monitoring station	Aerosol concentration range (mg/m^3)^	Particle size (µm)	Abbreviation used
SidePak8001 (AM510)	0.001–20	0.1–10	AQM1
SidePak8002 (AM520)	0.001–20	0.1–10	AQM2
DustTrak3601	0.001–150	0.1–15	AQM3

DustTrak and SidePak, both manufactured by TSI, are similar products that measure particulates based on light scattering laser-based monitoring of particles. The DustTrak has a bigger display panel touch-based selection and a larger concentration range. Based on previous research, we expected these AQMs to provide the appropriate measurement range (Jiménez et al., 2011).

The average and maximum values, and standard deviations, were compared between set ups (Table 3). Comparisons between the AQMs were limited in some cases due to the concentrations exceeding the upper monitoring limit (UML) of the monitors. AQM1 and AQM2 have a lower UML than AQM3 and were exceeded at multiple points. In test 2, where dung was burnt on the hearth, all three monitors were exceeded. Due to the maximum capacity of AQM1 and AQM2 being exceeded in some of the tests, the missing entries for these data points were replaced with the UML of the machines (20,000), prior to statistical analysis. As the AQMs ran for different lengths of time in each experiment, the period of comparison was set at 2 h 36 min for all experiments, to avoid for example misleading averages caused by an AQM continuing to collect data beyond the duration of the burning period. Statistical examination of the AMQ3 PM_2.5_ tests and statistical differences between tests have been assessed using Welch’s *t*-tests to account for differences in sample sizes and variance.

**Table 3:** Test 1 values recorded for 10 min

Test	Test 1			Test 2			Test 3			Test 4			Test 5		
AQM	1	2	3	1	2	3	1	2	3	1	2	3	1	2	3
Average	112	69	62	13,078	13,447	60,109	10,402	12,527	12,793	13,833	13,827	45,903	5192	5764	7689
SD	187	37	44	8006	8023	63,352	5965	6999	7943	4442	3761	44,887	5007	5395	7938
Max	585	112	185	19,771	19,914	150,000	18,351	**20,000**	25,300	19,540	19,070	149,000	19,733	18,115	36,900
Min	16	26	38	8	22	28	90	90	166	11	45	28	21	34	23

Tests 2–5 values calculated for time period of 2 h 36 min (full details of timing and duration of different experiments and AQM set-ups can be found in the Supplementary Data file)

Bold value indicates the upper monitoring limit (UML) of the monitor

## Results

**Fig. 3:**
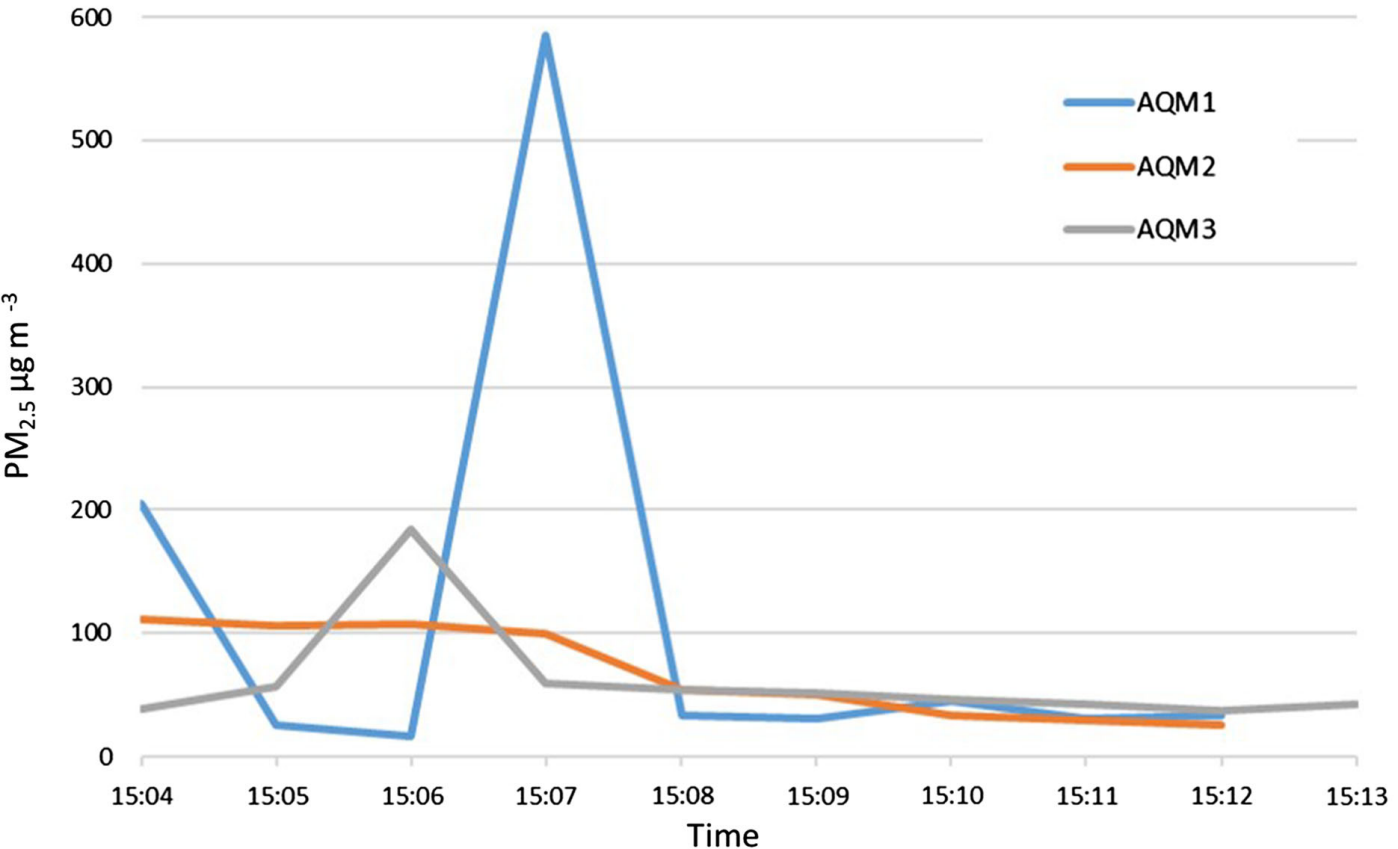
Background PM2.5 levels measured by the three air quality monitoring stations

In test 1, the background levels of PM_2.5_ in the building were recorded for 15 min for each AQM (Table 3, Fig. 3). The values ranged from 16 to 585 µg m^−3^ and averaged 62–112 µg m^−3^. Background levels were generally low, aside from the peak of 585 µg m^−3^ in AQM1. AQM2 and AQM3 also showed slightly elevated levels at the beginning of the measurement period. This may have been the result of localised stirring of dust on the floors during the equipment set-up, both from the soft nature of the plaster finish on the building floors, and from dust tracked in from the surrounding area.

### Test 2: Dung fuel on hearth

**Fig. 4:**
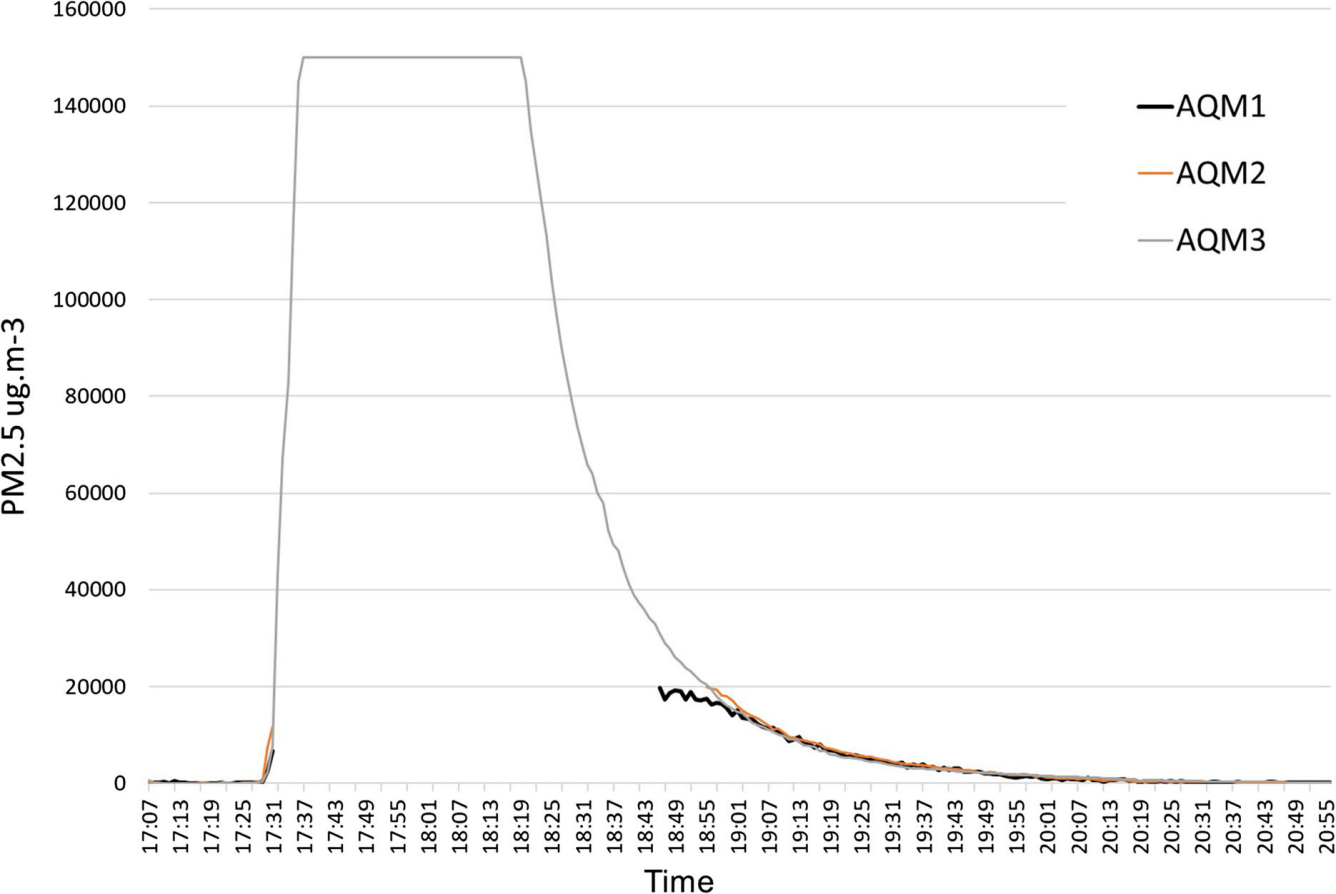
Test 2 showing PM_2.5_ concentrations from a dung fuel fire on hearth

In test 2, dung fuel was burnt on the hearth area in front of the oven. Figure 4 shows the time series data. PM_2.5_ values for the dung burning experiment climbed rapidly and exceeded the UML of all three monitors for around 1 h between 17:28 and 18:24. The highest level of 150,000 µg m^−3^ was recorded by AQM3, which is the UML of the machine. It is likely that the actual values were higher than this. The indoor pollution exceeded the maximum capacity of 20,000 µg m^−3^ for monitors AQM1 and AQM2 for the majority of the period. After 1 h, the PM_2.5_ levels detected by AQM3 declined rapidly from the maximum value but remained high staying in the range of 4000–2590 µg m^−3^ for another hour until the fire burned out completely.

### Test 3: Wood fuel in oven

**Fig. 5:**
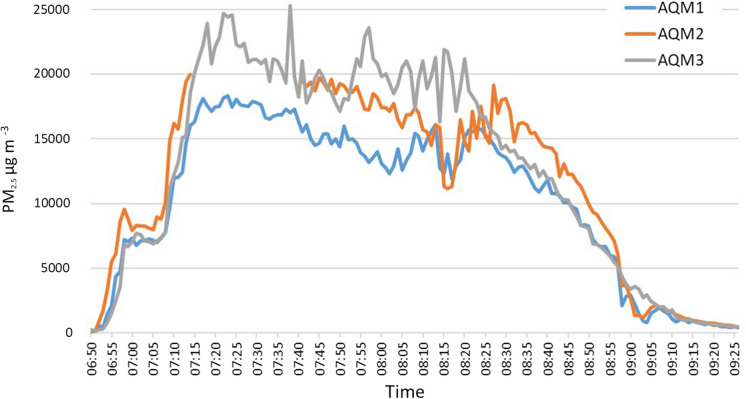
Test 3 showing PM_2.5_ concentrations from burning wood fuel in the oven

In Test 3 wood was burned for 2 h 36 min. Figure 5 depicts the time series of PM_2.5_. All monitors show a broadly similar pattern of concentrations, with a rapid, stepped increase to the maximum value after c. 20 min, remaining at this level for around 1 h before gradually decreasing over a further 1-h period, and a more rapid reduction for 45 min. AQM2 received highest particulate levels of 19,980 µg m^−3^ at 7:14, and AQM1 received a maximum value of 18,351 µg m^−3^ at 7:23. AQM3 recorded highest value of 25,300 µg m^−3^ at 7:38. The maximum levels surpassed the capacity of AQM2 for a period between 7.15–7.40 am, though given the overall trend in comparison with AQM1 and AQM3, this likely remained around 20,000 during this period. AQM2 was located further away but directly in front of the oven opening, whereas AQM1 was located to the side (Fig. 2).

The fire was extinguished at 8:20 at which point the particulate pollutant levels decreased until the end of the test, settling out after around 1 h. A slight upraised trend in the graph was likely due to the smouldering effect caused after the fire was extinguished.

### Test 4: Mixed fuel fire in oven

**Fig. 6:**
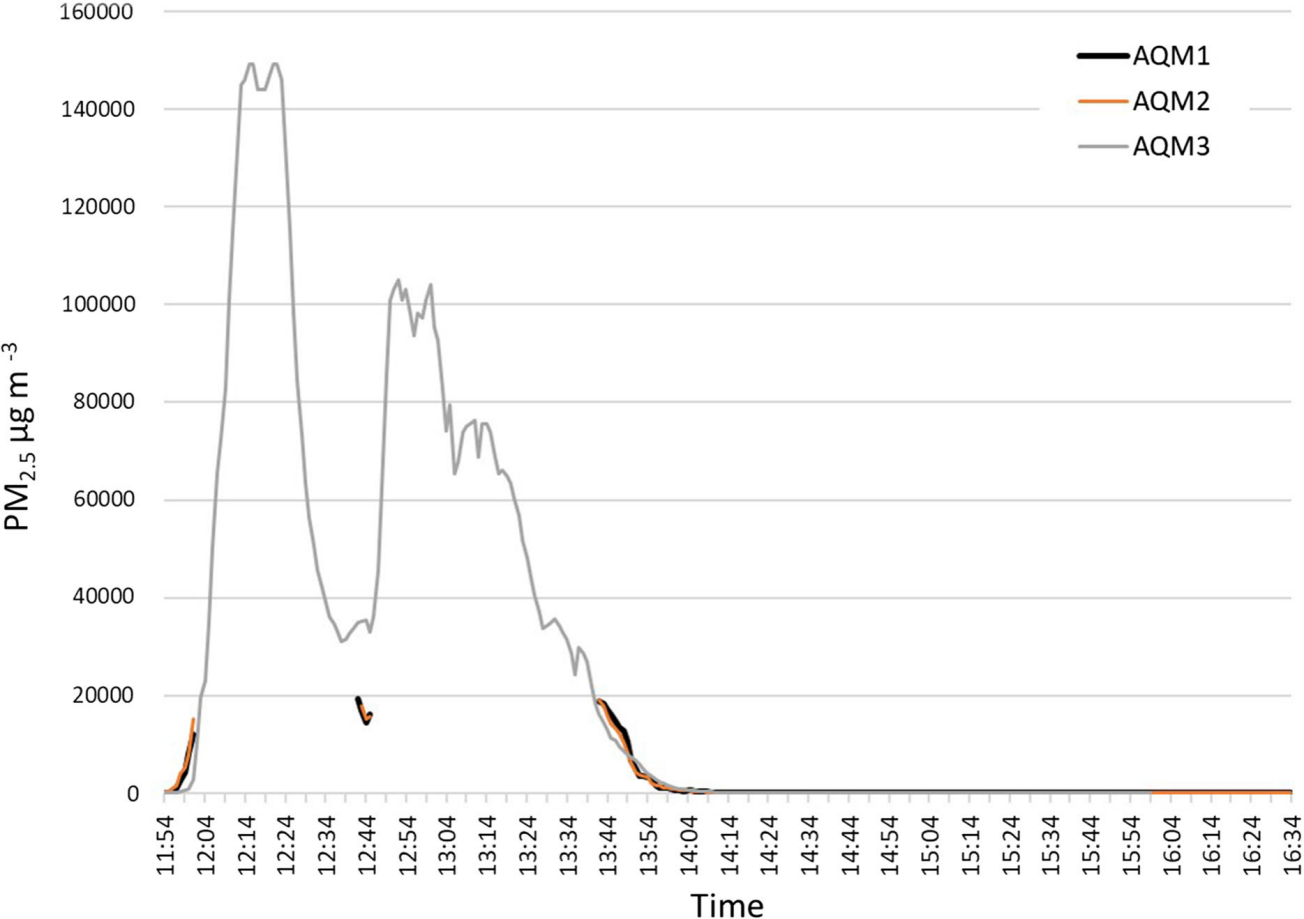
Test 4 showing PM_2.5_ concentrations from burning mixed fuel in the oven

Figure 6 shows the time series of PM_2.5_ in test 4. AQM1 and AQM2 were unable to capture the recordings where the pollution was greater than 20,000 µg m^−3^, which occurred for two periods, the first around 12:02 pm and continued for forty-two minutes, and the second around 12:47 pm for 1 h. The highest value attained by this test was captured by the two different monitors were 19,540 µg m^−3^ at 1:22 pm and 17,950 µg m^−3^ at 1:43 pm, respectively. As the concentration started to decrease after 1:44 pm, the period after this time was considered as the fire decaying period. While AQM1 recorded lowest value at the end of the study, the other monitor detected in the decaying time of the fire. AQM3 recorded a similar trend as the first two monitors; however, this monitor was once again able to record all the measurements due to its higher capacity (Figs. 8, 9). The concentrations initially followed the same pattern as the dung fuel in test 2 with a rapid increase to very high levels in PM_2.5_ achieving highest levels of 149,000 µg m^−3^ at 12:17 pm and 12:23 pm. This was followed by a drop off after 30 min, 20 min at a steady level around 20–30,000 µg m^−3^ with a second rapid increase, and stepped decrease. The overall duration of burning was around 2 h.

As Çatalhöyük is a World Heritage Site, it attracts a lot of tourists. It should be noted that Test 4 was disrupted for approximately 10 min when a tour group briefly opened the door of the experimental building, and some of the smoke is likely to have escaped from the outlet. This could explain the sudden drop observed in the concentrations, and the resurgence that followed after the door was closed.

### Test 5: Wood, refuelled

**Fig. 7:**
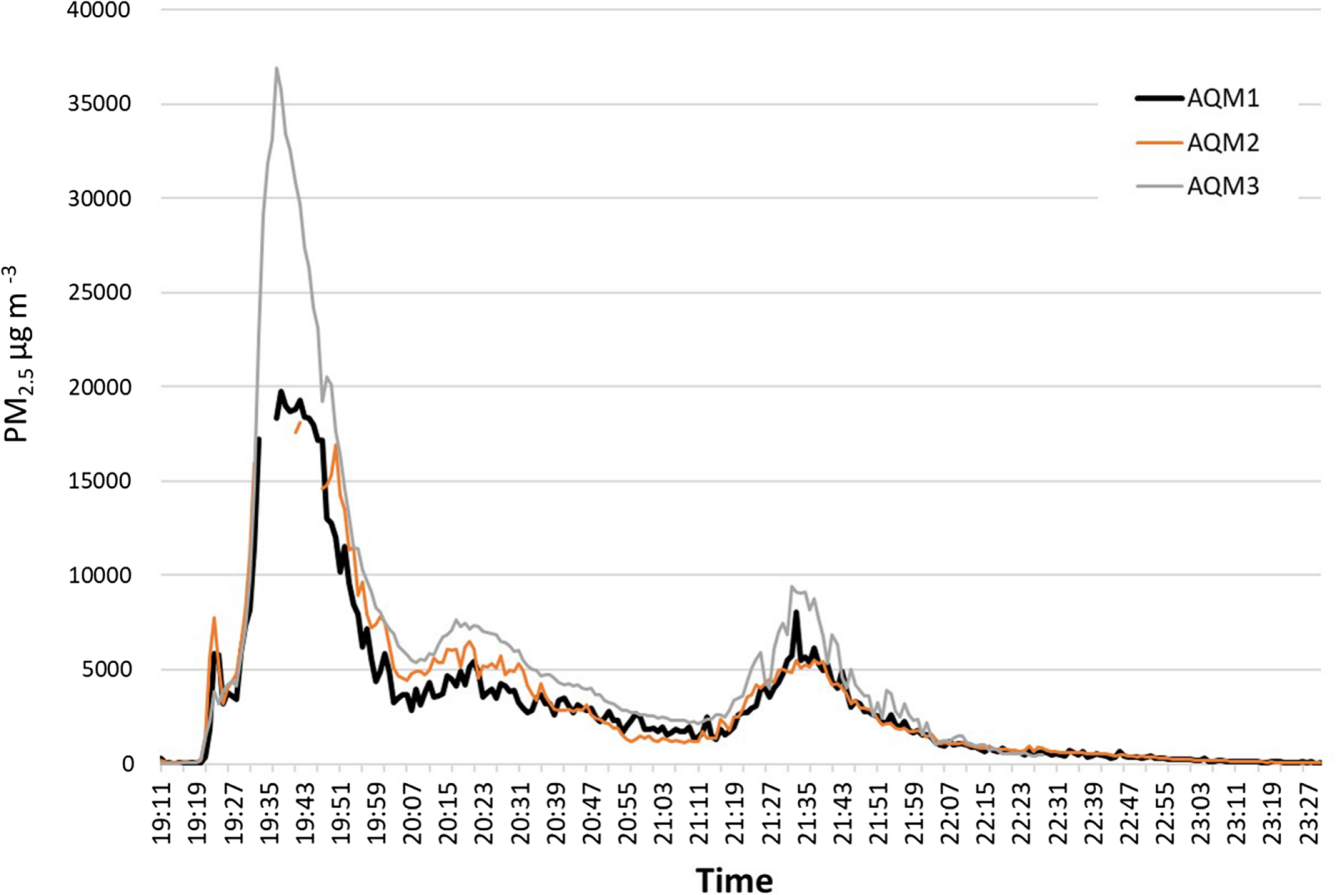
Test 5 showing PM_2.5_ concentrations from burning wood fuel in the oven, with a refuelling episode

The fire was ignited at around 19:19, and to increase its intensity, two wooden logs were added after 3 min. After this point, the pollutant level started to increase and AQM1 and AQM2 were unable to record the readings at 7:34 pm for 3 min as the quantity exceeded the instrument’s capacities. The peak value attained by this test was captured by the two different monitors were 19,733 µg m^−3^ at 7:39 pm and 18,115 µg m^−3^ at 7:43 pm (Fig. 7).

AQM3 recorded a similar trend as the first two monitors and this monitor was successful to capture all the PM_2.5_ quantities. After fire ignition at 7:19 pm, the levels escalated and reach a peak of 36,900 µg m^−3^ at 7:38 pm and then started to decrease rapidly. The peak values were detected for a period of 30 min, before settling in to a fluctuating level between 5–7000 µg m^−3^ for around 2 h followed by a gradual decline. The monitors were left to record the concentrations overnight after the fire had burnt out.

Results from the AMQ3 tests show that differences in fuel type and fuel location generated statistically significantly differences in PM_2.5_ concentrations (Table 4).

**Table 4:** Results from AMQ3 PM_2.5_ Welch’s *t*-tests

	Test 2	Test 3	Test 4
Test 2			
Test 3	*t*(241) = 1.97, < 0.01		
Test 4	*t*(412) = 1.97, 0.01	*t*(272) = 1.97, < 0.01	
Test 5	*t*(237) = 1.97, < 0.01	*t*(324) = 1.97, < 0.01	*t*(263) = 1.97, < 0.01

## Discussion

The first point to note is the background measurements. While these were very low in comparison with the fuel burning episodes, there is clearly a level of environmental input.

### Comparison between experiments and different fuel types

Tests 3–5 were conducted to compare the emissions from wood, mixed dung and wood, and wood refuelled, when burning in the oven. The maximum for all tests was in the region of 18–20,000 µg m^−3^ for AMQ1 and AMQ2, very close to the limit of detection of the monitors. The maximum recorded by AQM3 for tests 3, 4 and 5 was 25,000 µg m^−3^, 149,000 µg m^−3^ and 36,900 µg m^−3^, respectively (Table 3). All values are a similar order of magnitude aside from test 4 where dung and wood are mixed, when the concentrations are more than four times that of wood alone. The capacity of all three monitors was exceeded for at least part of the fuel burning episode in test 2, meaning the exact maximum figure is unknown, but in excess of 150,000 µg m^−3^.

**Fig. 8:**
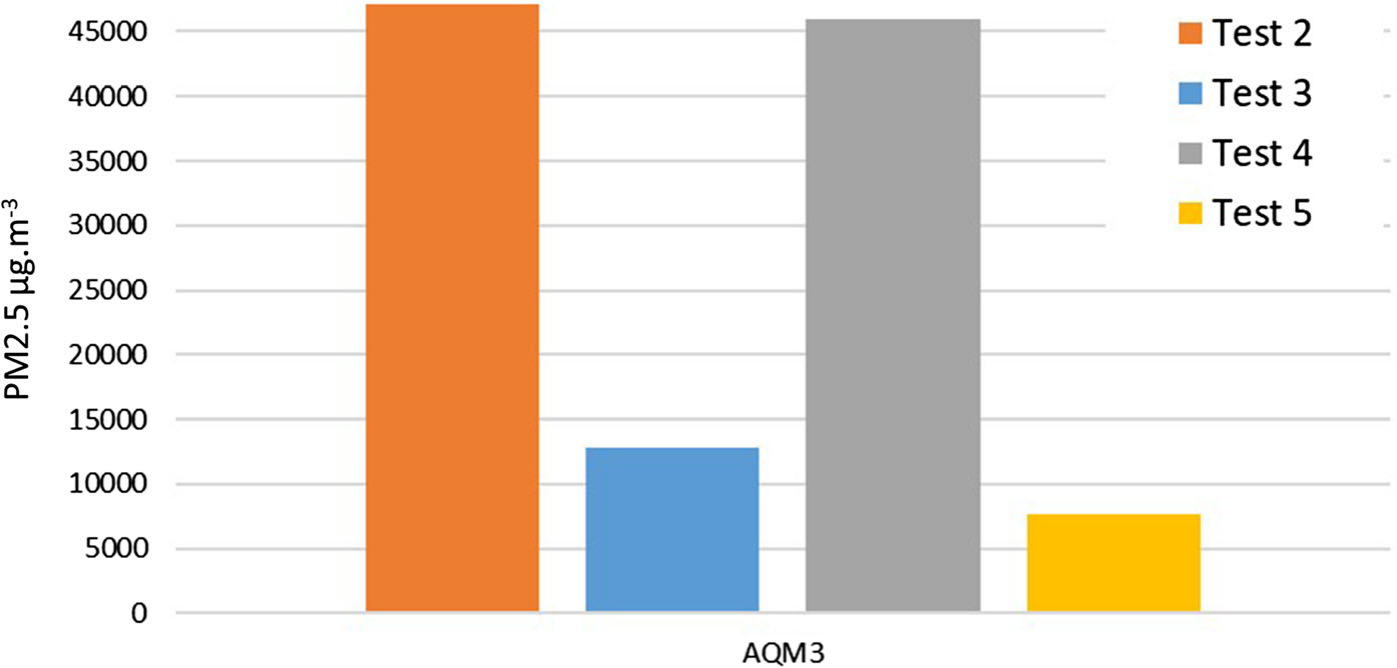
Average PM_2.5_ values detected over a 2 h period using AQM3

**Fig. 9:**
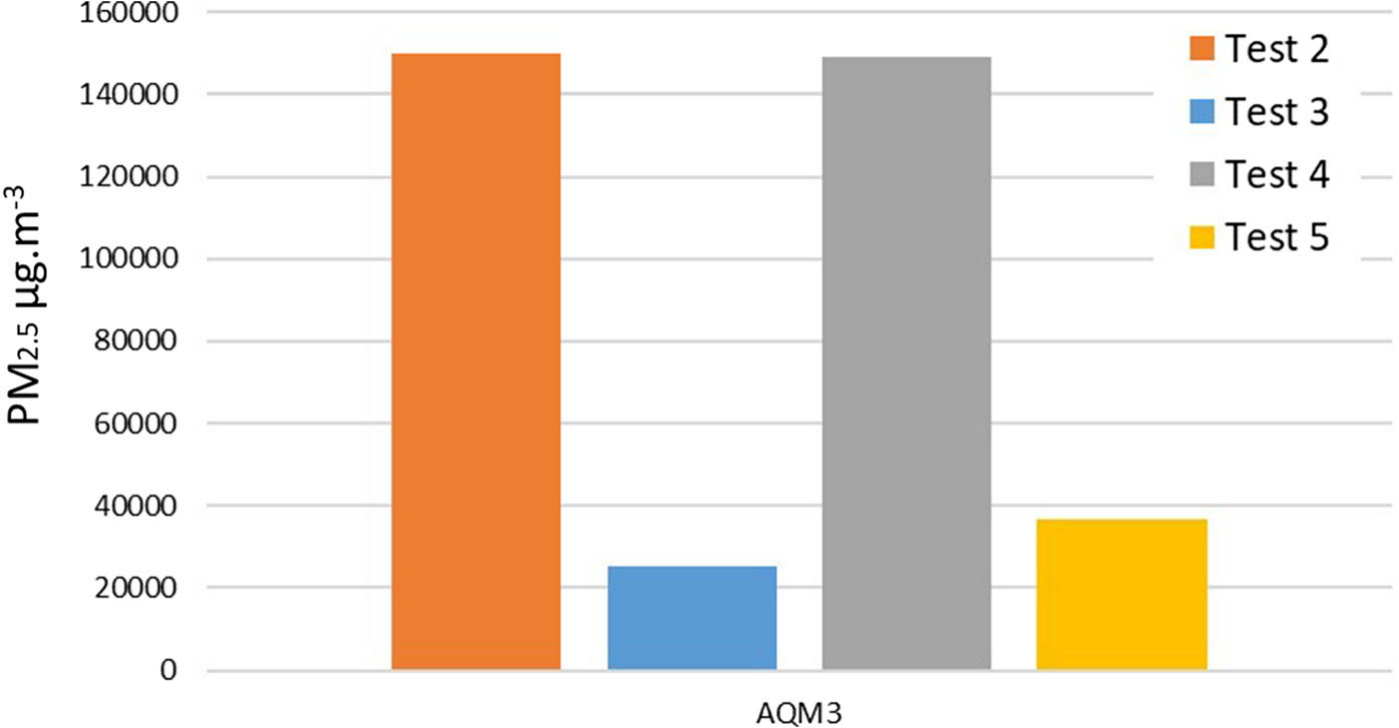
Maximum PM_2.5_ values detected by AQM3

AQM3 showed that test 2 containing dung as the fuel is the highest emitter of the PM_2.5_ pollution. This was followed by the test 5 comprising the mixture of wood and dung fuel, burnt in the oven. Statistical analysis indicates a significant difference between the different experimental set-ups (Table 4). The maximum for test 2 on the hearth was > 150,000 µg m^−3^ and test 5 was 36,900 µg m^−3^, suggesting that the oven did have a reducing effect on the emissions received by the AQMs, though it is also noted that the mix of fuel types may also account for a lower signal than when the dung was burnt by itself (Figs. 8, 9).

Dung was observed to be the highest producer of PM_2.5_ in this investigation, when compared with wood or mixed fuel, producing maximum values more than four times that of wood. This observation matches previously reported comparisons of dung and wood, for example, Joon et al. (2011) compared dung, wood and other fuels burnt in traditional and modern stoves in India. In their study, the maximum values from dung were 11,000 μg m^−3^ and averaged 774 μg m^−3^ over 24 h, compared with wood at 223 μg m^−3^. Dung mixed with wood (test 4) showed similar high levels of PM_2.5_ even when this was burnt inside the oven rather than outside the oven on the hearth. Wood as the sole fuel source (test 3 and 5) produced lower levels of PM_2.5_ but still at levels much higher than the background levels.

### The impact of house structure and ventilation

It is known in modern studies of air pollution that building configuration and ventilation play a key role in determining levels of exposure to fuel burning by-products (Balcan et al., 2016). Multiple studies indicate that domestic heating is a greater cause of pollution than industry, particularly during winter due to people spending more time indoors with fires for heating (Mudway et al., 2005; Özden et al., 2008; Taşdemir et al., 2005). The structure of buildings at Çatalhöyük is unusual from a modern perspective in that no evidence for windows or doors has been uncovered. It is thought that inhabitants entered and exited the building through an opening in the roof. A typical house at Çatalhöyük had a domed oven set against the south wall, located beneath an opening in the roof. The way the ovens functioned is unclear—there is no archaeological evidence for chimneys or flues, and multiple attempts at lighting fires in the experimental house inevitably have resulted in the building becoming rapidly filled with smoke (Eddisford et al., 2009; Shillito et al., 2017).

A similar situation was observed when Skov et al. (2000) conducted experiments in a reconstructed a Danish Iron Age structure to examine indoor air pollution. In this structure, a similar type of thatched roof was present, and the outlet for the smoke created was through the thatched roof and louvres in the ridge and the gables. In both Çatalhöyük and the Danish Iron Age structure, the infrastructure of the house is likely to play an important role. Both are enclosed with no structure for ventilation.

In comparison, for another fuel burning experiment at the Lejre Centre in Denmark, researchers carried out a similar experiment in reconstructions of seventeenth–nineteenth century farmhouse buildings. In these buildings, the hearths were located in the middle of the building with total dimensions of 13 × 3.7 m, ceiling height of 1.9 m. In this case, a 6 m high chimney was also present. At Lejre, the PM_2.5_ had a daily average 138–1650 µg m^−3^ inside the hearths and 21–160 in the adjacent living rooms. Calculated daily exposure for individuals in close proximity to the hearth averaged 196 μg m^−3^ of PM_2.5_. (Rhyl-Svensen et al., 2010). Measurements of air distribution at Lejre showed that the chimneys created a draw effect that quickly removed the smoke from the hearth area, thus high levels of exposure would only have impacted individuals in very close proximity to the fire. Interestingly, the PM_2.5_ levels near to the fire were still lower than those observed in our study, or by Skov et al. (2000).

In our study, we focused analysis on the 2–4 h period when the fire was actively burning, to avoid distorting the average by including a long ‘tail’ of background measurements. In test 5, the monitors were left running for 10 h, some 7 h after the fire burnt out. If the full duration of measurements over the 10 h is included, the average reading for that day would be 1336–1839 μg m^−3^. At Çatalhöyük, the lack of a proper chimney, and that fact that buildings consist of a single small room that combined living space and the hearth, means that all inhabitants would have been exposed to these high levels, not just those working in close proximity to the fire.

In our experiments, concentrations continued to remain high up to 40 min after the fires had burnt out afterwards. Test 3 indicates greater exposure directly in front of the oven, though overall similar levels to the side of the opening, suggesting that the relative position of a person in relation to the fire had only a minimal impact on exposure. The positioning of all the three monitors influenced the variations observed in the results, with individuals who spent periods of time in closer proximity to the fire were at greater risk; however, the small size of the rooms meant that the area of close proximity covered most of the living area, and although there was some variation between monitors, levels were extremely high in all AQM positions.

### Implications for respiratory health at Çatalhöyük

From our observations, it is almost certain that burning fuel indoors at Çatalhöyük exposed inhabitants to unsafe levels of particulates. The observations in this experiment, therefore, raise a number of interesting questions concerning fuel use in the Neolithic settlement, and the impact this would have had on the health of the inhabitants. Early studies of human skeletal material at Çatalhöyük identified black carbon residues on the interior surface of ribs from three individuals, which was interpreted as evidence for anthracosis (Andrews et al., 2005; Birch, 2005). Upon re-examination, however, it is debatable whether these black deposits represent in vivo accumulation of soot within the lungs or are post-depositional in nature. In any case, the number of individuals affected by these residues is very low.

The low number of individuals with unequivocal skeletal indicators of respiratory disease contrasts with our observations; however, there are some osteoarchaeological observations that could relate to chronic lung disease. Two studies by Larsen et al., (2015, 2019) review the health of the population over its 1000+ year occupation from an osteoarchaeological perspective. In the Early period (7100–6700 cal BCE), the population was small and hypothesised to consist of only a few households, and grew to perhaps 3500–8000 individuals during the Middle period (6700–6500 cal BCE), the peak population size. These estimations are based on the number and size of houses (Cessford, 2005). In the Late period (6500–5950 cal BCE), the population appears to have decreased; buildings were more dispersed, and there was more open space than in previous periods.

Osteoperiostitis is a generalised inflammatory response to infection and is present in the Çatalhöyük assemblage, and largely associated with lower limbs (Larsen et al., 2019). There is a significant change over time, with the population showing more osteoperiostitis in the Middle period, with a rapid decline in the Late period (prevalence is 33–26 to 19% in the Early, Middle, and Late periods, respectively Larsen et al., 2019: 6). In terms of age-related patterning, a previous analysis revealed that sub-adults show the highest levels in the Early period, a slight decline in the Middle period, and a greater decline in the Late period (Larsen et al., 2015: 54). In the sub-adult category, neonates and infants have a considerably higher prevalence of osteoperiostitis, which likely represents this age group’s increased risk for infection as a result of their immature immune systems.

Both children and adults appear to have had adequate diets, with few signs of nutritional stress (Larsen et al., 2015: 50). The population does show an elevated prevalence of dental caries, which is linked to consumption of carbohydrates (Larsen et al., 2015: 58). Periostitic lesions are therefore unlikely to relate to nutritional stress, given that all other indications suggest good availability of food. Larsen et al. (2019) attribute periosteal reactions to increased population density, which is believed to have promoted the transmission of pathogens and increased the likelihood of tissue infection from cuts; Larsen et al. (2019) suggest the prevalence of subperiosteal lesions on tibial diaphyses in farmers compared to foragers globally is a result of localised bacterial infection. We suggest perhaps this is also linked to chronic smoke exposure, as farming communities spent extended periods indoors compared to foraging communities.

One difficulty is that osteoperiostitis is non-specific so cannot be clearly linked to a specific infection or disease, but given the indirect evidence from our experiments, an additional explanation for these observations could be that the periosteal lesions are a result of chronic exposure to PM_2.5_. The inhalation of PM_2.5_ is a hazard because the small particles can travel deep into the lungs where they become embedded in the tissue and can even enter the blood stream, triggering an inflammatory response outside the lungs. In modern studies, increased bone deposition on long bones has been linked specifically to lung disease (West, 2012).

Exposure to PM_2.5_ is also linked to increased susceptibility to infection, and it could be that these factors are both linked to the bone lesions. Staphylococcus aureus, for example, a commonly occurring skin bacteria, is known to be altered by exposure to black carbon in smoke, and the particulates can transfer the bacteria from the nasopharynx to the lungs (Hussey et al., 2017). As well as the direct adverse effects of indoor air pollutants, exposure can also lead to a weakened immune system and increased vulnerability to respiratory tract infection, and other indirect impacts such as low birth weights (Kurmi et al., 2010). Although it is not possible with the current data to make a direct link between the osteoarchaeological record and respiratory disease attributed to fuel burning, our experimental work strongly suggests this possibility, if we assume that people were spending time indoors on a regular basis.

## Conclusions

This is the first study to examine particulate concentrations in an early prehistoric settlement. All of the burning activities within the experimental Çatalhöyük house created unsafe levels of PM_2.5_, with the use of dung either by itself or mixed with wood, having around four times higher levels of PM_2.5_ than wood alone. Even though the pollution created by the wood was comparatively less than that of the dung, the PM_2.5_ pollution produced by both fuels overall exceeded the European standard limits and the WHO limits of 25 µg m^−3^ and 20 µg m^−3^, respectively, by many orders of magnitude. The lack of openings or chimney meant little ventilation was present, leading to an accumulation of particulates.

PM_2.5_ in modern contexts leads to direct and indirect health impacts, and increases susceptibility to other disease. If people were burning fuels regularly indoors in the Çatalhöyük buildings, they certainly would have been exposed to extremely high levels of unsafe PM_2.5_, and extended exposure would have had an impact on respiratory health. It may be expected that such an impact would be detected in the human remains; however, clear osteological evidence is lacking. While several non-specific indicators are observed in published studies, some of which could potentially be linked to lung disease, preservation issues and their non-specific origins make it difficult to be certain whether these bony lesions are associated with pulmonary disorders or other types of disease. Long bone periostitis and arthritis observed in the human remains have previously been attributed to general infection and workload. We propose that these may also be linked to lung disease through chronic exposure to exceptionally high levels of PM_2.5_, in combination with increased susceptibility to infection.

Understanding the exposure–response relationship (i.e. the relationship between differing levels of exposure to air pollution and the actual incidence of disease) is difficult in modern contexts due to the short term nature of most studies (Ezzati & Kammen, 2002). While the relationship between biomass burning and increased indoor air pollution is well known, the mechanisms are poorly understood. There have been limited studies on the impacts of exposure to sustained high levels of air pollution over long time periods, on health and mortality (Huang et al., 2018).

The extended chronology of the archaeological record has the potential to help understand the long-term (> lifetime) relationship between health and the built environment, and the potentials are just beginning to be explored (Shillito et al., 2017). Collaborative research involving archaeology and environmental engineering offers a new approach to studying such questions. Future research will expand these experiments to investigate a wider range of scenarios.

## Supplementary Information

Below is the link to the electronic supplementary material.

Supplementary file1 (XLSX 127 KB)
